# Rare genetic E196A mutation in a patient with Creutzfeldt–Jakob disease: a case report and literature

**DOI:** 10.1080/19336896.2020.1769528

**Published:** 2020-06-05

**Authors:** Xiping Wu, Zhao Cui, Xie Guomin, Haifeng Wang, Xiaoling Zhang, Zhiguang Li, Qi Sun, Feiteng Qi

**Affiliations:** Department of Neurology, Ningbo Medical Center Li-Huili Hospital, Ningbo, China

**Keywords:** Creutzfeldt–Jakob disease, mutation, PRNP, E196A

## Abstract

Genetic Creutzfeldt–Jakob disease (gCJD) is characterized by mutations in the PRNP gene and represents approximately 10–15% of the human prion diseases. Here, we report a 42-year-old Chinese man who was diagnosed with gCJD. The patient had a rare mutation in codon 196 (E196A) of PRNP leading to an exchange of amino acid from glutamic acid (E) to alanine (A). The polymorphism of codon 129 in the patient was methionine homozygote. His mother and daughter are asymptomatic carriers of the same mutation. The clinical manifestations were similar to those of sporadic CJD. 14-3-3 protein was positive in cerebrospinal fluid, and there were sharp slow complex waves in electroencephalography and ribbon-like signals on magnetic resonance imaging (MRI). The main complaints of patient changed from visual space and visual colour to psychotic symptoms with enhanced high signal intensity on the occipital and frontal cortices on MRI. We compared the clinical characteristics of the current patient with those of previously reported Chinese patients with other gCJD of E196A mutation to summarize the common features of E196A gCJD.

## Introduction

Prion disease or transmissible spongiform encephalopathy (TSE) is a rare and fatal neurodegenerative disease. At present, it is believed that the pathogenic mechanism of TSE is abnormal accumulation of misfolded prion protein (PrP^sc^) in the central nervous system. Human TSEs include Creutzfeldt–Jakob disease (CJD), fatal familial insomnia (FFI), Gerstmann–Sträussler–Scheinker syndrome (GSS) and Kuru disease. According to the aetiology, CJD is classified as four subtypes: sporadic, genetic, iatrogenic and variant [1]. About 85% of all CJD cases are sporadic, 10–15% are genetic and less than 1% is acquired [2].

Human genetic prion diseases are caused by mutations in the prion protein (PrP) encoding gene PRNP which is located on the short arm of chromosome 20 [3]. More than 60 point or insertion mutations in the PRNP have been associated with human genetic prion diseases. Among these mutations, D178N FFI, T188K genetic CJD (gCJD), E200K gCJD, P105L GSS and E196A gCJD were top five in China [4]. T188K gCJD is the most common PRNP mutation in the Chinese population. E196A gCJD is very rare and has been reported in four patients in China [2–5]. Here, we reported a 42-year-old male patient with a heterozygous mutation in codon 196 (E196A: c.587A>C, GAG-GCG). The clinical characteristics of this E196A gCJD patient are similar to those of the sporadic CJD.

## Case report

A 42-year-old male office worker was admitted to our hospital with complaints of progressive dementia, metamorphopsia and change in visual space and visual colour for 2 months. He could not know the direction when driving a car. He found all cars were the same grey when parking. He had no medical or psychiatric records, previous surgeries, blood transfusion or hormonal injection. He did not take any conventional drugs. No relevant family history was reported. Neurologic examination revealed cortical blindness in both eyes, an increase in the muscle tone of the four extremity, hyperreflexia, paroxysmal tremor of the four limbs, instability of finger–nose experiment and heel–knee–shin experiment. He could only move with the assistance of one family member due to ataxic gait. He had no ability to complete complex task such as drawing a full clock. MMSE (Mini-mental State Examination) scored 24 points (college culture). The patient underwent obviously rapid cognitive decline. By day 14 since admission, akinetic mutism, severe agitations and myoclonic jerks were observed. By day 30 after admission, psychic hallucination and delusion were developed. The patient discharged automatically.

Basic full blood count, electrolytes, liver and renal functions, vitamin B_12_ level and blood sugar level were normal. Thyroid function test with anti-thyroid peroxidase was normal. C-reactive protein and erythrocyte sedimentation rate were normal. Antinuclear antibody, extractable nuclear antigen, anti-neutrophil cytoplasmic antibodies, anti-double-stranded DNA (dsDNA), serum VDRL and HIV were all normal. Anti-NMDA receptor (NMDAR) antibody, anti-gamma-aminobutyric acid-B receptor (GABABR) antibody, anti-leucine-rich glioma-inactivated 1 (LGI1) antibody, anti-contactin-associated protein-like 2 (CASPR2) antibody, anti-α-amino-3-hydroxy-5-methyl-4-isoxazolepropionic acid 1 (AMPA1) receptor antibody, anti-α-amino-3-hydroxy-5-methyl-4-isoxazolepropionic acid 2 (AMPA2) receptor antibody, anti-dipeptidyl peptidase-6 antibody (DPPX), anti-IgLON family member-5 antibody (IgLON5), anti-glutamic acid decarboxylase antibody (GAD65) and antimetabolic glutamate receptor-5 (mGluR5) were all negative in both serum and cerebrospinal fluid (CSF). Lumbar puncture showed normal opening pressures, with CSF findings as follows: nucleated cells 1 × 10^6^/L and protein 22.2 mg/dl. Other CSF tests for bacterial disease, tuberculosis, cryptococcal antigen, fungal disease and viral encephalitis (herpes simplex virus, varicella-zoster virus, Epstein–Barr virus and cytomegalovirus) were normal.

**Figure 1.**
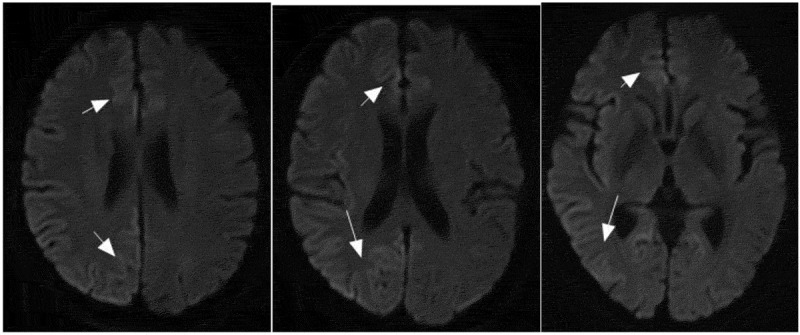
Ribbon-like high signal intensity in the frontal, occipital and parietal cortices in the right hemisphere.

**Figure 2.**
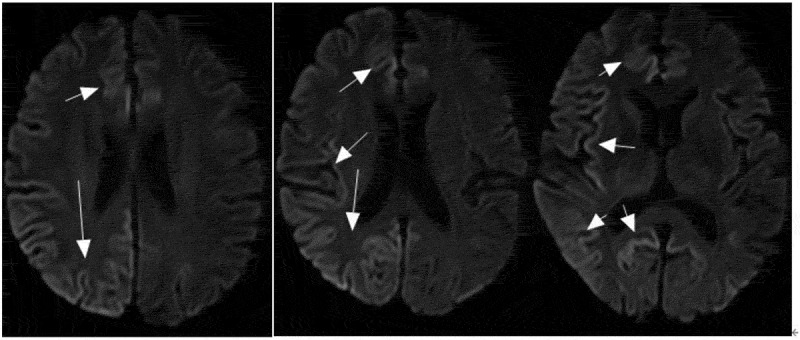
Ribbon-like high signal intensity in the frontal, occipital and parietal cortices enhanced.

**Figure 3.**
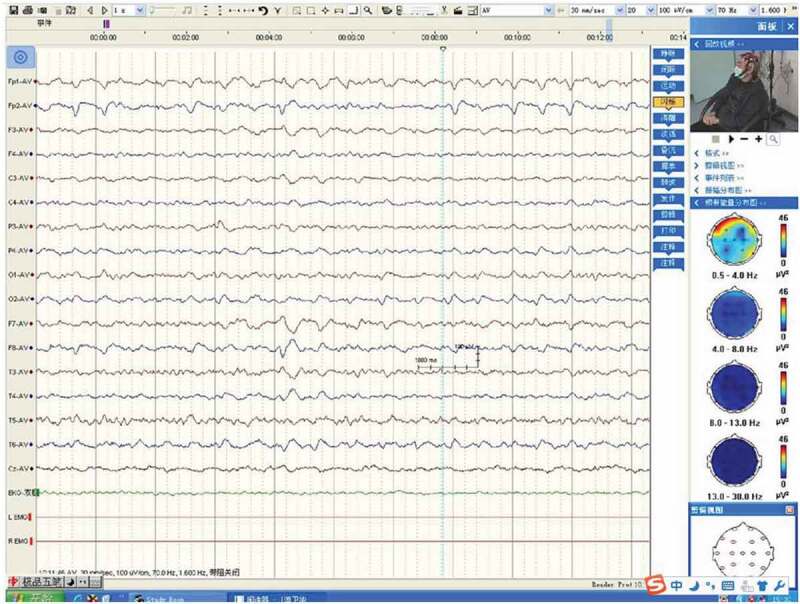
Sharp slow complex waves in the first EEG.

**Figure 4.**
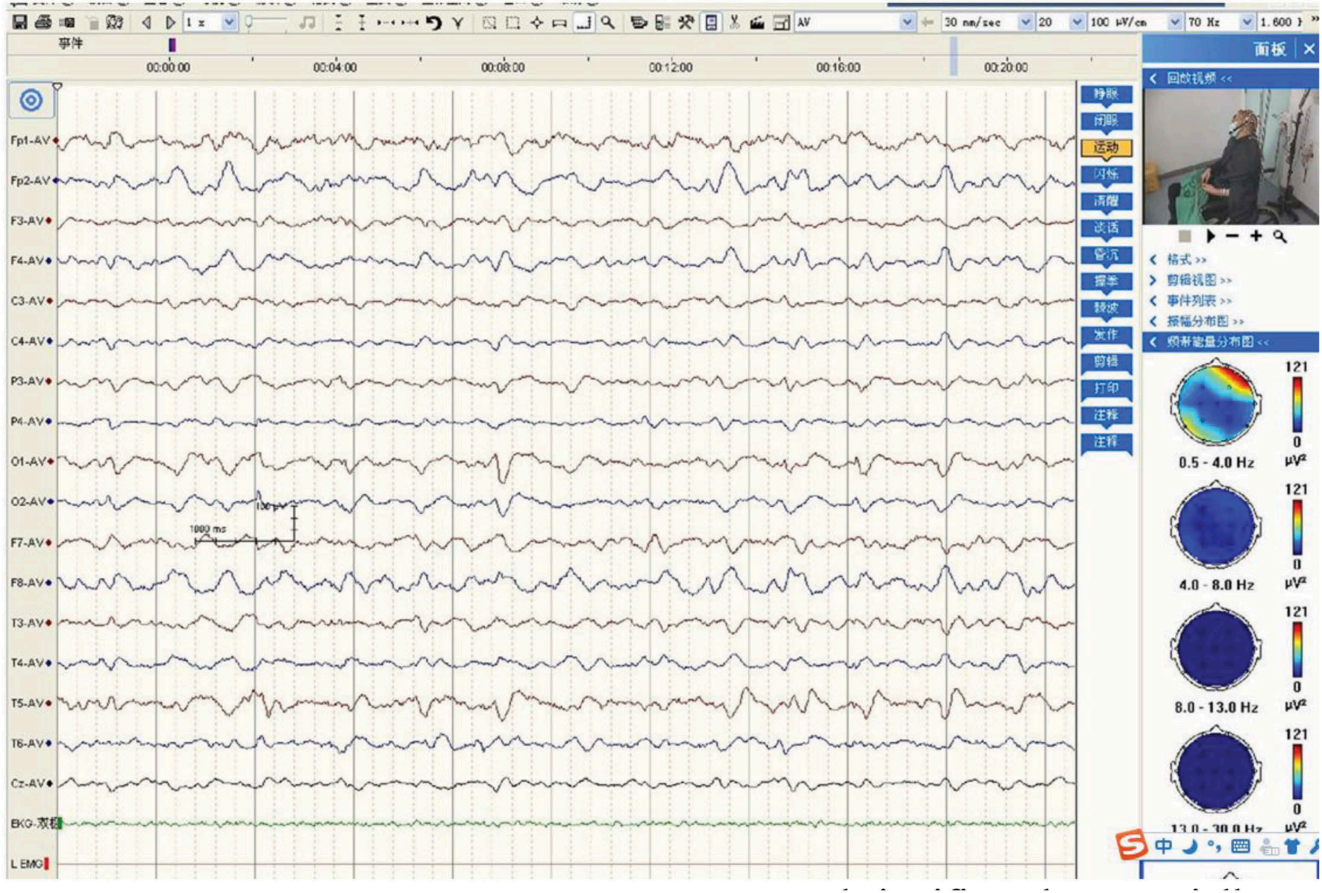
Sharp and slow complex waves increased significantly, especially in the right frontal lobe and left rear head.

**Figure 5.**
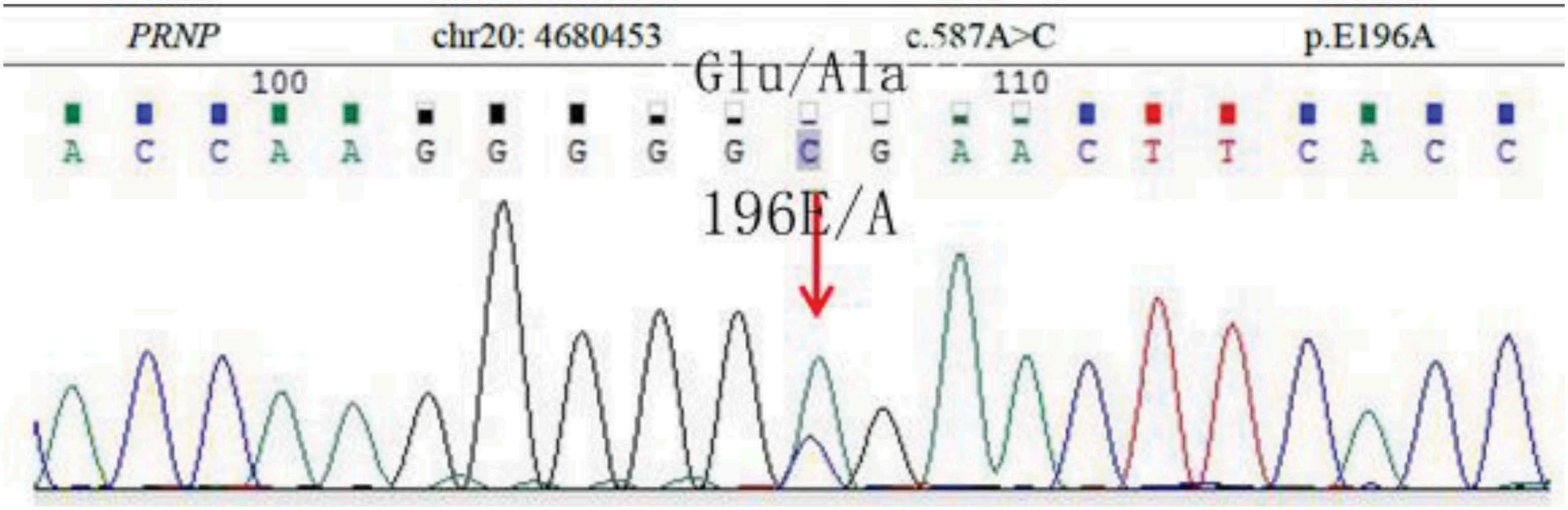
DNA sequence of the patient shows an E to A heterozygous transition at codon 196.

**Figure 6.**
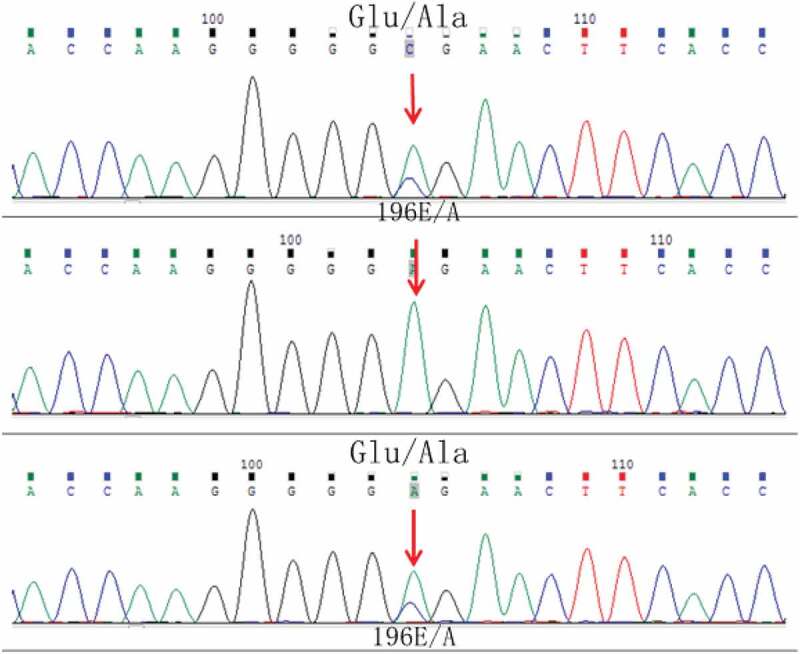
DNA sequence of the PRNP gene in parents and daughter of the patient shows an E to A heterozygous transition at codon 196 on the first line (his daughter) and the third line (his mother) of the picture.

Magnetic resonance imaging (MRI) diffusion-weighted imaging (DWI) revealed ribbon-like high signal intensity in the frontal, parietal and occipital cortices in the right hemisphere (Figure 1). After intravenous immunoglobulin and antiviral therapy, with the development of the psychic hallucination of the patient, ribbon-like high signal intensity in the frontal, parietal and occipital cortices enhanced on MRI (Figure 2). The 14-3-3 protein was found in the CSF. There were sharp slow complex waves in electroencephalography (EEG) (Figure 3), and after half a month, sharp and slow complex waves increased significantly (Figure 4). A heterozygous missense mutation in PRNP gene, c.587a > C, p.e196a, was found via next-generation sequencing and sanger sequencing (Figure 5). The polymorphism of codon 129 in the patient was methionine homozygote. His mother and daughter were asymptomatic with the same mutations in codon 196 (E196A) of PRNP, and the polymorphism of codon 129 of them was all methionine homozygote(Figure 6).

## Discussion

Our patient is very young (42 years old). According to the report, the mean age of gCJD is 30–55 years old which is younger than that of sCJD (55–75 years old), and the duration of gCJD is comparatively longer than that of sCJD (usually <1 year) [2]. The onsets of the patient are visual space and visual colour change which are typical occipital lobe symptoms. This phenotype is similar to a Heidenhain variant of CJD. Heidenhain variant of CJD is a peculiar clinical presentation of sporadic CJD characterized by isolated visual disturbances at disease onset and reflecting the early targeting of prions to the occipital cortex [6,7]. A patient with nucleotide substitution of an isoleucine for a valine at codon 180 (V180I) in PRNP also presented with visual hallucinations and illusions [6]. The disease onset of different gCJD patients might resemble a Heidenhain variant of sCJD. From Table 1, The initial symptoms of the previous four patients were different greatly from our patient. But with the development of the disease, the manifestations of these patients were more and more similar to the typical symptoms associated with sCJD, including rapid progressive cognitive decline, myoclonus, cerebellar ataxia, pyramidal and extrapyramidal symptoms, akinetic mutism and psychiatric symptoms. According to the previous report, the histological changes, PrPSc type and clinical features of gCJD patients with methionine homozygote at codon 129 are overall very similar to those of the sCJD methionine homozygote phenotype [8], which explains why the five E196A gCJD patients had almost all the typical symptoms of sCJD.

**Table 1.** Clinical characteristics of the five patients with E196A gCJD.

	Case1	Case2	Case3	Case4	Case5
Age at onset	76	55	57	56	42
Gender	Male	Female	Female	Female	Male
Duration (months)	10	22	NA	NA	NA
Familial history	-	-	-	-	-
Initial symptoms	Intellectual decline and intermittent mental and behavioural disorders	Persistent decline in intelligence, giddiness and unsteady walk	Dysarthria and weakness of the left hand	Numbness and weakness of the left limbs, obvious dizziness and paroxysmal jitter of the left limb	Intellectual decline, visual space and visual colour change
Clinical features	+	+	+	+	+
Cerebellar ataxia	+	+	+	-	+
14-3-3 Protein in CSF	+	+	+	+	+
MRI (DWI) (ribbon-like signals)	+	+	+	+	+
EEG	PSWCs in EEG	Bilateral diffuse waves, occasionally with periodic sharp waves	Moderate abnormality especially in the right hemisphere	A lot of abnormal waves in the background, including sharp waves, sharp slow waves and slow waves	A lot of sharp slow waves
Codon 129	MM	MM	MM	MM	MM

NA: not available; clinical features include: rapidly progressive dementia, myoclonus, visual problems, pyramidal symptoms, extrapyramidal symptoms, akinetic mutism and psychiatric symptoms; CSF: cerebrospinal fluid; MRI: magnetic resonance imaging; DWI: diffusion-weighted imaging; PSWCs: periodic sharp wave complexes; MM: methionine–methionine.

In a large Indian family with familial Creutzfeldt–Jakob disease, genetic analysis displayed a D178 N mutation in two symptomatic individuals and seven asymptomatic members [9]. In a Chinese report, 21 members from 3 families implemented PRNP sequencing, and 16 unaffected carriers of E200K mutation were found [10]. From the report mentioned above, we can conclude that although gCJD is a dominant genetic disease, the penetrance is not high. The mother and daughter of our patient had the same mutations in codon 196 (E196A) of PRNP, but they were asymptomatic.

CSF 14-3-3-positive rates in the patients with E196A gCJD were relatively high [4]. The 14-3-3 protein was positive in CSF in all five E196A gCJD patients given in Table 1 [4]. Periodic sharp wave complexes (PSWCs) had been revealed in about two-thirds of patients with sCJD. But in gCJD, topical PSWC could be seen in less than 20% patients (Table 1). Patients with gCJD usually exhibit diffuse, non-specific sharp waves, sharp slow waves and slow waves [2,3,9]. The patient’s characteristics are consistent with the above-mentioned reports. MRI can detect the characteristic sCJD changes with 91% sensitivity and 95% specificity on DWI sequences [1]. With the development of the psychic hallucination of the patient, ribbon-like high signal intensity in the frontal, parietal and occipital cortices enhanced on MRI. But some report displayed no significant change in the pattern and distribution of cortical ribboning on repeated MRI despite severe clinical deterioration observed [1].

It has long been noticed that 129 M/M homozygotes are associated with sCJD and vCJD patients [2]. 129 M/M homozygotes increase the frequencies of sCJD. The evaluation of methionine homozygotes at codon 129 among the prion disease patients in a research cohort revealed the increased frequencies of sCJD compared to the general population [11]. 129 MV heterozygosity provides relative protection against sCJD [12]. In all five E196A gCJD cases, the PRNP polymorphisms of codon 129 were all 129 M/M, which indicates that 129 M/M may be associated with E196A genetic mutation. But more cases are needed to prove the relationship between E196A mutation and the 129 M/M polymorphism.

A family with genetic prion disease produced a psychological burden. The decision to accept the test does not relieve people’s anxiety about family diseases. gCJD is a dominant genetic disease, but the penetrance is not so high. Genetic counselling procedures should consider all these situations and help to relieve the psychological burden of the patients’ families, even that of noncarriers and that of untested [13].

In conclusion, we report a rare young patient who carried codon 196 (E196A) mutation of PRNP. His asymptomatic mother and daughter had the same mutations. Compared with the other patients, this case provided further knowledge of E196A gCJD to better understand the diagnosis and research of gCJD.
